# History of traditional Mongolian medical education based on *Manba Datsan*: A literature review

**DOI:** 10.1002/hsr2.1702

**Published:** 2023-11-21

**Authors:** Buyandelger Batmunkh, Munguntuul Enkhbat, Taivanjargal Gankhuyag, Oyunaa Chantuu, Oyungoo Badamdorj

**Affiliations:** ^1^ School of Nursing Mongolian National University of Medical Sciences Ulaanbaatar Mongolia

**Keywords:** historical periodization, *Manba Datsan*, medical education, philosophical underpinnings, traditional Mongolian medicine

## Abstract

**Background and Aims:**

Traditional Mongolian Medical Education (TMME) was developed based on *Manba Datsan (MD)* in harmony with the Buddhist philosophy‐medical system in Mongolia. It was developed intensively during the 17th–20th centuries and was interrupted for a while, but it is still a part of medical care in Mongolia, training traditional medicine doctors and nurses. Its historical roots are inextricably linked with medical and philosophical development not only among Mongolians, but also in some Oriental and Western countries. This review aims to raise awareness and promote TMM, following the WHO guidelines on traditional medicines.

**Methods:**

Relevant literatures from Google Scholar, PubMed, Mongolian national and university libraries in the past 30 years were collected in this article, and books, of which the history, philosophy, and culture of TMM were analyzed. We used inductive analysis within the constructivist paradigm and conducted the research as follows: (I) analyze relevant literature reviews; (II) formulate the historical periodization of Mongolian medical education (MME); (III) explore an overview of the philosophies that have been influenced by TMME; and (IV) study the contents and phases of *MD* training in Mongolia.

**Results:**

An integrated version of the historical periodization of the MME was developed with fully compatible historical periodization based on important socio‐political turning points in Mongolia. TMME has been clarified by the direct and mutual influence of Oriental, and Western medical concepts. Between the 17th and 20th centuries, TMME was developed based on *MD*. Since 1990, the training process has been conducted in accordance with the international standards of modern vocational and higher education.

**Conclusion:**

Traditional Mongolian Medical Education was formed by underpinnings of the achievements of Mongolian national practical and folk medicine. This process was greatly influenced by the traditions of the Mongolian education system and achievements of oriental medical education.

## 1 INTRODUCTION

The concept of Traditional Mongolian Medical Education (TMME) has not been studied and published worldwide. The TMME can be considered the official formulation of medical education (ME) within the Tibetan Buddhism prevalent in Mongolia. Formal education system was developed between the 17th and early 20th centuries based on *Manba Datsan (MD)*. This institution has provided Traditional Mongolian Medical (TMM) knowledge and skills (Tibetan language, basis of Buddhist philosophy, four medical tantra, etc.,) for discipleship, and it is called *Manramba* (nowadays physician), who earned the proper skills and sufficient knowledge of healing people. In addition, they informally trained ordinary people in Mongolian practical and folk medicine content. Currently, the formal TMME is provided by medical universities with traditional medicine and the traditional education organization based on *MD* is transferred for religious purposes.

TMM is an independent system that has philosophies, history and culture and it is separate from Mongolian history and culture. The findings revealed that the traditional treatments from China, India, and Tibet have been influenced,^1^ and developed among different Mongolian ethnicities.^2^ TMME successfully integrated the progress of oriental ME with the cultural and ethnographic characteristics of Mongolians. Therefore, we use the principle of cultural relativism^3^ to understand TMME. Ethnographic studies of ME based on sufficient evidence are few,^4^ but they can generate valuable insights.

## 2 METHOD

We used the ethnographic research method in this review and searched the related literature. The ethnographic research method is includes an analysis content of meaning, process, and context^5^ and is demonstrated in tables and figures. In TMME's ethnographic research, it is very appropriate to conduct an inductive analysis that is coordinated with the development process of Mongolian history, culture, and philosophy within the framework of the constructivist paradigm. Relevant literatures from Google Scholar, PubMed, Mongolian national and university libraries in the past 30 years were collected in this article, and books, of which the history, philosophy, and culture of TMM were analyzed. This research carried out the following processes: (I) analyze relevant literature reviews; (II) formulate the historical periodization of MME; (III) explore an overview of the philosophies that have influenced TMME; (IV) study the contents and phases of *MD* training in Mongolia. Our results expressed the three main factors of history, philosophy, and culture that influence traditional medicine practices.^6^ We used the following methods such as, for historical periodization were used concept of important turning points^7^; and for philosophical underpinnings were used the “five color states” model^8^; and for cultural factors were formulated from Datsan which on based Buddhist education, respectively.

## 3 RESULTS

### 3.1 Historical roots of TMME

Clarifying the historical roots of TMME is a research process that provides evidence of events that have already occurred in Mongolians before the formation of TMME, and develops a concept of how it has progressed. In historical research, it is optimal to develop historical periodization based on important turning points, in addition to explaining the phenomenon under study in a simple chronological order. The content of each period and stage of historical periodization should be clear and, better connected, but not too complicated.^9^ There have been some attempts to develop a historical periodization of the MME, but most have been developed by relating the development of Mongolian medicine or education to ideological and socio‐political processes.^10^, ^11^, ^12^, ^13^, ^14^, ^15^, ^16^ These historical periodizations are based on some important turning points in the social and political history of Mongolia, but they are not fully consistent with the current model, and they have not yet been able to fully express the pattern of the MME's development by considering only medicine and education. In the last 10 years, the concept of Mongolian historical periodization has been updated,^17^ therefore, we have analyzed the previous versions and developed an integrated version shown in Table 1.

**Table 1:** Integrated version of the Traditional Mongolian Medical Education (TMME) historical periodization.

Author	Year	Focus	Period and stage of Mongolian history^18^										
			Ancient period		Medieval period					Modern period			
			Prehistory (before 3rd century BC)	Ancient states (3rd century BC–10th century)	Khamag Mongol (11th–12th century)	Great Mongol (1206–1260)	Mongolian Empire (1260–1368)	Political dissolution (end 14th century to beginning 17th century)	Mongolia under Qing Empire (17th‐beginning 20th century)	Early 20th century (1911–1923)	Socialist Mongolia (1924–1990)		Democratic development (since 1991)
Khaidav^19^	1997	Ideology and medicine	Shamanist ideology (Early Mongolian medicine)				End of shamanist ideology		Tibetan Buddhist ideology		Russian ideology		Modern medicine
Batkhuyag and Batdelger^20^	1997	Education and politics, economy	Early Mongolian Education			Feudalistic education		End of feudalistic education	Education of Qing Empire	Autonomic Mongolian education (1911–1921)	Revolutionary education (1921–1960)	Socialistic education (1960–1990)	Market economy
Nyamdavaa^21^	2001	Medicine and ideology	Practical medicine		Folk medicine		Traditional medicine		Oriental medicine		Western medicine	Socialist ideology	Democratic ideology
Natsagdorj^22^	2003	TMM	Origin of TMM (before 12th century)			Development of TMM (until 16th century)			Diffusion of TMM (16th century –1937)			Interruption of TMM (1937–1990)	Recovery of TMM
Shagdar^23^	2009	Education and politics	Ancient education (before 13th century)			Mongolian Empire, and posteducation (13th–17th century)		Qing Empire stage	Liberation revolution stage	Independently education (1921–1940)	Formal education	Democratic education (since 1987)	
Lkhagvasuren and Oldokh^24^	2011	Medicine and education	None	Folk MME					Influence of oriental ME (16th‐19th century)	*Datsan*‐based MME	Western ME based on Oriental ME (1921–1942)	University‐based MME	International standard ME
Bold^25^	2023	Medicine and ideology	Practical medicine (before 209 BC)	Folk medicine (209 BC‐1206)		Complex of oriental medicine (1206–1578)			TMM (1578–1921)		Western medicine (1921–1940)	Socialist ideology (1940–1990)	Reform of TMM

Integrated version of the MME historical periodization													
			**Ancient period MME**		**Medieval period MME**					**Modern period MME**			
Recommended version		Mongolian history, medicine and education	Prehistory of MME	MME of ancient states	Influence of oriental ME (11th–16th century)				Manba Datsan ‐ based TMME (17th–20th century)	Influence of Western ME (Early 20th century)	Vocational school‐based MME (1929–1942)	University‐based MME (1942–1990)	Modern MME (since 1990)

Archeological findings of acupuncture therapy and, trepanning were found in Mongolia approximately 4000 years ago.^26^ During the ancient kingdoms, treatment methods such as bloodletting therapy (*khanuur zasal*) and moxibustion (*toonuur zasal*) matured^27^ and had famous doctors,^28^ which indicating that the basis of practical MME may have been formed. A feature of the ancient period of the MME was its evidence‐based, practice of animal treatment,^29^ some of which methods have been handed down to this day.^30^ Subsequently, in the 5th century, Buddhism spread as the religion of the royal court,^31^ which created preconditions for the penetration of some results of oriental ME in Mongolia.

In the 13th and 14th centuries, under the influence of the Mongolian campaigns in several directions of Eurasia, the form of informal education within the framework of the military organization of the MME, teaches the methods of treating and nursing injuries such as wounds, broken bones, dislocations, burns, and blows, has developed intensively.^32^ At this time, the vocabulary of the Mongolian language includes *emchi* (who treats with drugs), *otochi* (who treats with herbs and plants), *bariachi* (who treats concussion), *domchi* (who treats with exceptional skills), *sharkhachi* (who provides wound healing care).^33^ Some researchers found and noted that following historical activities such as medical school that began in 1261,^34^ the Indian, Chinese, and Arab medical achievements were introduced.^35^ As a result, doctors become a famous, one of them was *Hu Sihui*.^36^ From the beginning of the 17th century, when the Manchu‐Mongolian monarchical union was established,^37^ an oriental ME tradition integrated with Tibetan Buddhism formed the official Mongolian ME system based on *MD*.

**Figure 1:**
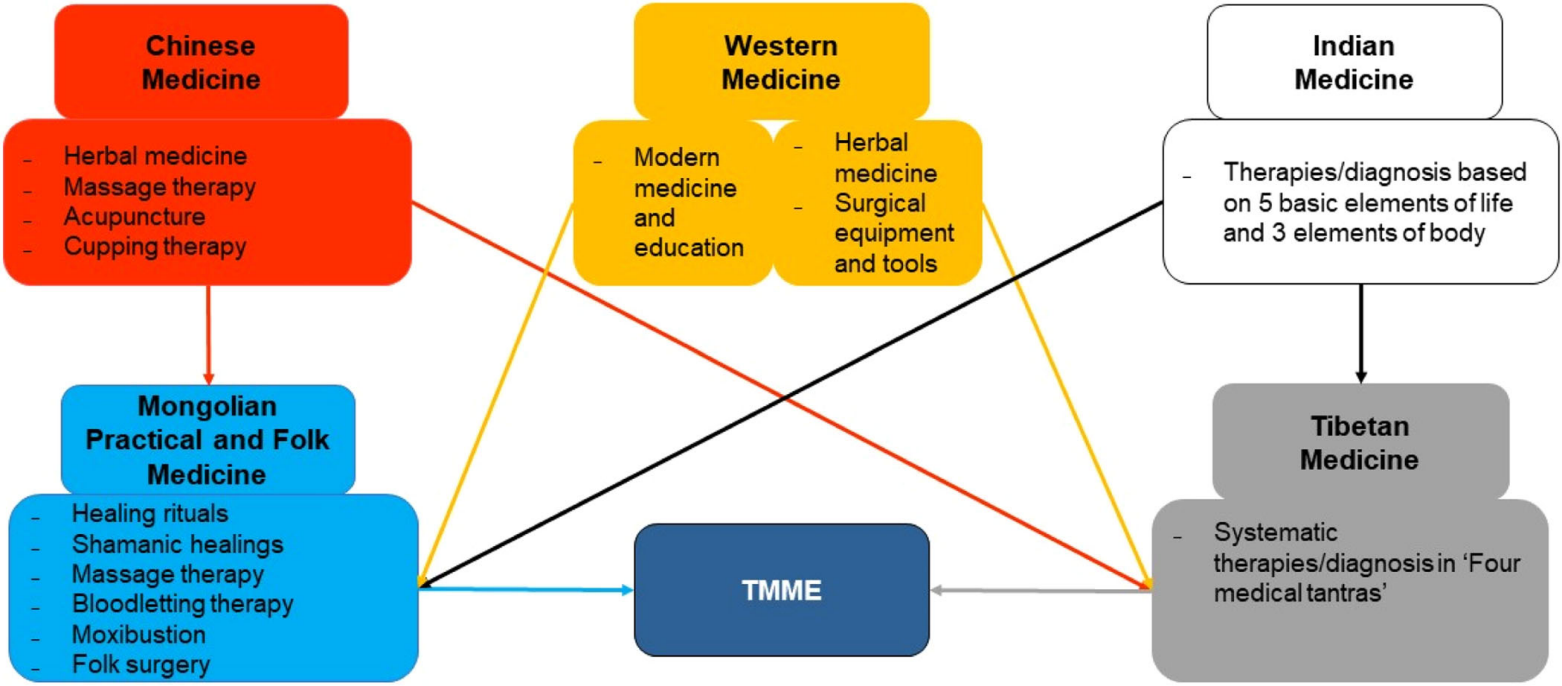
Overview of main therapies/diagnosis Influencing Traditional Mongolian Medical Education (TMME). Based on the traditional Mongolian “five color states” model, how Oriental and Western main therapies/diagnosis influenced the formation of TMME is outlined. Mongolian and Tibetan medicine, the two primary sources of TMME, have creatively developed Chinese, Indian, and Western therapies/diagnosis traditions. However, it is difficult to accurately show the changes in historical time. For example, the main therapies/diagnoses of China and India, which influenced Mongolian Practical and Folk Medicine, belonged to the time before the 17th century, while the modern medicine and education system was influenced by Russia in the 20th century.

According to historical evidence and literature, the development of discipline for TMME requires the inheritance Mongolian culture, knowledge, experience, and striving into tradition and innovation. Besides, it is to continuously influence scientific knowledge and achievements from external sources such as India, Chinese, and Tibetan main therapies. TMME characteristics were developed, such as learning from the past and building the present and the future by inherited into their own traditions. It has been reported^38^, ^39^ that this process continues and develop. According to Figure 1, the main therapies and diagnosis of Indian and Chinese medicine are directly and indirectly (through Tibet), Greco‐Arabic medicine indirectly, and Western modern medical and educational systems have been directly influenced by Russia. Mongolian healing rituals (dom zasal), shamanic healing (*böögiin zasal*), massage therapy (*baria zasal*), bloodletting therapy (*khanuur zasal*), moxibustion (*töönuur zasal*), and folk surgery (*mes zasal*) are the main therapies and diagnosis of TMM, and they are still preserved today.

### 3.2 Philosophical underpinnings of TMME

**Figure 2:**
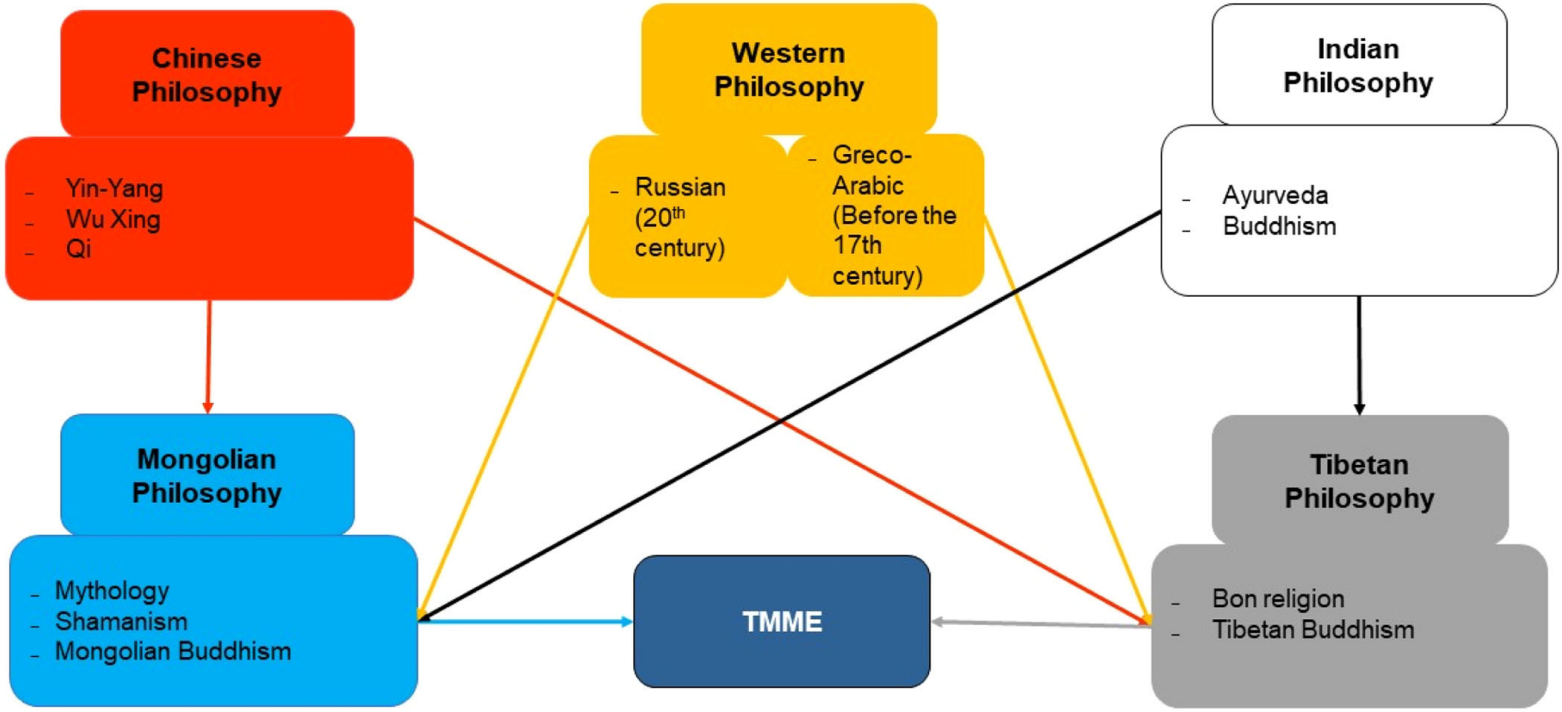
Overview of Philosophical Concepts Influencing Traditional Mongolian Medical Education (TMME). TMME was based on *Manba Datsan*, and followed the philosophical ideals of Mongolian Buddhism. However, the main therapies/diagnoses of the Oriental and West reflect the influence of the philosophical concepts of these countries. Using the Mongolian traditional 'five color states' model, philosophical concepts that directly or indirectly influenced TMME are shown in here. It does not clarify the interaction of the philosophical concepts of the above countries, but only aims to show the influence of TMME in a one‐way flow.

Medical education deals “with human life and well‐being, and requires special knowledge, skills, and behavior.”^40^ Therefore, to fully understand the concept of TMME, it is appropriate to clarify the Mongolian philosophical underpinnings and the influence of Oriental and Western philosophy. Five philosophical‐medical traditions (Mongolian, Chinese, Tibetan, Indian, and Western) influenced the formation of TMME. The results are shown in Figure 2.

During the prehistory of the MME, mythology prevailed as the main basis of philosophy^41^ and tradition with a nonscientific logic based on healing rituals. This ideology of Mongolians was not immediately lost in the course of further development, and it has been an important basis for the development of ways to prevent and fight diseases based on the similar symptoms of human and animal diseases.^42^ Subsequently, the scope of the MME within Shamanism^43^ expanded and came to represent the character of a traditional discipleship (*shavi surgalt*) associated with a rigidly established that one must master to become a shaman. However, since this system is not a form of professional training focused only on the acquisition of medical knowledge and skills, it was an alternative form of MME. Shamans did not give specific names to diseases (typhoid, scabies, etc.), but generally formulated them as “filth (*buzar*),” “sufferings (*gai bartsad*)”^43^ and treated them in the form of shamanic healings.^44^ “Sunesu” (combination of soul and spirit) was the main concept of MME with shamanism, and this concept is fundamentally different from the concept of “soul” in the Western philosophical tradition.^45^ The Shamanist MME framework underpins one of the most enduring ideologies in the history of the MME.

From the time of the Xiongnu Empire, the principles and conceptions of Chinese philosophy, such as *Yin‐Yang* and *Wu Xing* ^46^ influenced MME and formed a combined pattern. However, it is difficult to say that the above principles and concepts originate only from China, and they are widely spread in Asian peoples, not only in medicine, but also in the metaphilosophical level. For example, the Mongolian *Arga‐bileg* model not only has the same meaning as the Chinese *Yin‐Yang* principle, but is also deeply embedded in Mongolian culture and thought.^47^ These concepts have remained an important component of TMM, even under the strong influence of Buddhism.^48^ Buddhism was introduced to Mongolia from India in the 5th century, and later from Tibet in the 13th and 17th centuries,^49^ resulting in the formation of the core of TMME. Five basic elements of life and three elements of the body of Ayurveda spread through Buddhist concepts became the core teachings of TMME. Tibetan Buddhism combines the traditions of Ayurveda with the philosophical‐medical systems of the Chinese, Greco‐Arabic, and Tibetan Bong religions.^50^ However, it cannot be denied that the concept of Ayurveda has spread among Mongolians, not only from Tibet.^51^ Russian ideology was directly and strongly influenced by the socio‐political imperatives of the 20th century, leading to the disruption of the official TMME system.

### 3.3 TMME based in *MD*

As part of the process of spreading Buddhism in Mongolia, the official education system of TMME based on *MD* was established. *Datsan* was an organizational form of education in four main fields^52^: Philosophy, medicine, astrology, and crafts, in addition to being dedicated to Buddhist rituals.^53^ The first *MD* in Mongolia was established in 1585^54^ and by the beginning of the 20th century, there were more than 120 MDs in Mongolia.^55^ Fewer children were admitted to the *MD* because of traditional *shavi surgalt*. *MD*'s training graduated for about 16 years and taught the Buddhist conception and knowledge, skills of TMME. A system was created to invite famous doctors and teachers from countries with similar ME training, such as India and Tibet, or to visit these countries to improve their profession.^56^

The *MD* training was carried out in four phases according to the “four medical tantras” to acquire TMME.^57^ Since the Tibetan language was the main language of education, it was one of the special features of *MD* in Mongolia that the “preparatory phase” was added to teach students the language, and to familiarize them with the basic concepts of Buddhist philosophy (Table 2). Traditionally, *Yuthok Yonten Gonpo*'s “Four Medical Tantras”^58^ has been used as a textbook. Also, by using volumes 206–210 of “*Tanjur*,” which includes the medical content of Ayurvedic text, and medical works written by Mongolian monks in Tibetan language,^59^ in addition to ME from oriental countries, Mongolian practical and folk medicine was continuously inherited.

**Table 2:** Traditional Mongolian Medical Education (TMME) training based on *Manba Datsan*.

Level of training	Brief description	Basic content of training
Preparatory phase^60^	A phase to teach children to read and write and improve memory	Tibetan language
		Memorization of Lamaist texts
		Basic Buddhist philosophy
Root tantra^61^	A phase providing an overview of the entire treaty	Condition of the body
		Symptoms of the disease
		General understanding of treatment
Explanatory tantra^61^	A phase describing in detail the human body, health, death, medicines, diagnosis, and instrument	Embryology
		Anatomy
		Physiology
		Health
		Signs of death
		Disease (causes; modes; classifications)
		Behavior and diet
		Diagnosis
		Therapeutics
		Medicines (tastes; potency; compounding)
		Medical instruments
Instructional tantra^62^	A phase relating to the causes, symptoms, and treatment of various diseases and disorders	Diseases (humorial; upper body; internal organs; miscellaneous; caused by spirits)
		Disorders
		Sores
		Wounds and lesions
		Poisons
Subsequent tantra^63^	A phase of where diagnostics and pharmacology are studied in depth	Pulse and urine diagnosis
		Calming and cleansing medicines
		External treatments

Boys are enrolled at the age of about ten, the preparatory phase takes 4–6 years, and “four medical tantras” training takes 5–9 years.^64^ This variation in graduate years is related to the student‐learning skills. *MD*‐based TMME is unique in that there is a male predominance among students, which is rooted in some features of Tibetan Buddhism.^65^ The basic principles of teaching are to endure “Three bad habits of the learner or *Saviin gurvan gem*” (mouth facing down; impure; hole in the bottom) and “six values of learning or *zurgaan khuran medel*” (thinking of oneself as a patient; imagining the teacher as a doctor; viewing books as medicine; comparing learning with healing; respecting the theory and teaching; realizing the need for knowledge) is focused on shaping.

From the middle of the 19th century, Russian physicians provided long and short‐term medical care in Mongolia^66^ and exerted an appropriate influence on TMME, but they could not change its system. However, according to the decision of the 6th Congress of the People's Republic of Mongolia^67^ held in 1930, the government will no longer officially finance Mongolian‐Tibetan medical aid. In the future, a strict line will be drawn for the development of European medicine. In line with this, the *MD*‐based TMME was interrupted, and some of the people who had acquired the TMME through *MD* were persecuted and executed, while a small group secretly continued to provide care to the people and maintain their traditions. However, in 1960, the Institute of Mongolian Folk Medicine was established, and within its framework, the foundations were laid for the revival of TMM with new modern contents and methods. Subsequently, in 1990, the activities of *MD* began again, but the training process was carried out in accordance with the international standards of modern vocational and higher education.

## 4 CONCLUSION

TMME was constructed by underpinnings of the achievements of the Mongolian national practical and folk medicine. This process was greatly influenced by the traditions of the Mongolian education system and achievements of oriental ME. In the long period from the 3rd century BC to the 16th century AD, Mongolians were able to form a unified philosophical‐medical version as a result of intellectual and cultural relations with oriental and neighboring countries. The study of the history of the *MD*‐based system of TMME from the 17th to the 20th centuries is of great importance when there are initiatives to redevelop MME based on national concepts.^68^ When the TMM reaches the nowadays, it has already reached a version that adequately reflects the achievements of modern science in the West, and not only in a based on the Buddhism. Thus, the TMME system should have a proper combination of tradition and innovation.

## AUTHOR CONTRIBUTIONS

**Buyandelger Batmunkh**: Conceptualization, methodology, visualization, writing—original draft, writing—review & editing. **Munguntuul Enkhbat**: Conceptualization, methodology, software, visualization, writing—original draft, writing—review & editing. **Taivanjargal Gankhuyag**: Methodology, resources, writing—original draft. **Oyunaa Chantuu**: Resources, software, writing—original draft. **Oyungoo Badamdorj**: Conceptualization, supervision, writing—review & editing.

## CONFLICT OF INTEREST STATEMENT

The authors declare no conflict of interest.

## TRANSPARENCY STATEMENT

The lead author Buyandelger Batmunkh affirms that this manuscript is an honest, accurate, and transparent account of the study being reported; that no important aspects of the study have been omitted; and that any discrepancies from the study as planned (and, if relevant, registered) have been explained.

## Data Availability

All authors have read and approved the final version of the manuscript had full access to all of the data in this study, and take complete responsibility for the integrity of the data and the accuracy of the data analysis.
